# Extended active inference: Constructing predictive cognition beyond skulls

**DOI:** 10.1111/mila.12330

**Published:** 2020-12-02

**Authors:** Axel Constant, Andy Clark, Michael Kirchhoff, Karl J. Friston

**Affiliations:** ^1^ Charles Perkins Centre The University of Sydney Sydney New South Wales Australia; ^2^ Culture, Mind, and Brain Program McGill University Montreal Quebec Canada; ^3^ Wellcome Centre for Human Neuroimaging University College London London UK; ^4^ Department of Philosophy The University of Sussex Brighton UK; ^5^ Department of Informatics The University of Sussex Brighton UK; ^6^ Department of Philosophy Macquarie University Sydney New South Wales Australia; ^7^ Department of Philosophy University of Wollongong Wollongong New South Wales Australia

**Keywords:** active inference, affordances, cognitive niche construction, ecological psychology, extended mind, predictive processing

## Abstract

Cognitive niche construction is the process whereby organisms create and maintain cause–effect models of their niche as guides for fitness influencing behavior. Extended mind theory claims that cognitive processes extend beyond the brain to include predictable states of the world. Active inference and predictive processing in cognitive science assume that organisms embody predictive (i.e., generative) models of the world optimized by standard cognitive functions (e.g., perception, action, learning). This paper presents an active inference formulation that views cognitive niche construction as a cognitive function aimed at optimizing organisms' generative models. We call that process of optimization extended active inference.

## 1 INTRODUCTION

This paper reviews generic predictive approaches to niche construction to propose a specific model of cognitive niche construction under active inference. We clarify the mechanics of some important components of the cognitive niche that have yet to be addressed under active inference, namely, the functional and psychological components. We then argue for a view of extended active inference (henceforth, extended active inference [EAI]) based on our model of cognitive niche construction. This introduction provides a definition of the key concepts we refer to in this paper and outline of the proposed argument.

### 1.1 Concepts

#### 1.1.1 The cognitive niche

In cognitive science, cognitive niche construction can be viewed as a form of instrumental intelligence whereby organisms “create and maintain cause–effect models of the world as guides for prejudging which courses of action will lead to which results” (Tooby & DeVore, 1987, p. 2010). For instance, juvenile Capuchin monkeys zero in on stones proper to nutcracking activity by relying on traces left behind by experienced Capuchins. Residues are left on sites where successful nutcracking activity took place, which indicates to newcomers that stones found on those sites are suitable for nutcracking (Fragaszy, 2011). Traces, stones, and dispositions to social learning here form the ingredients of the cognitive niche as a cause–effect model.

The concept of the cognitive niche employed in cognitive science refers to the concept of the developmental, “ontogenetic niche” (West & King, 1987). The concept of the developmental niche asks a set of questions different from that of the selective niche (Stotz, 2017); it asks question about “not what's inside the genes you inherited, but what the genes you inherited are inside of” (Stotz, 2010, p. 1). This set of questions is especially interesting to study the epigenetic and behavioral sources of variations upon which selection can act. In turn, the concept of the selective niche is well suited to study the manner in which selection pressures are transformed by organisms. In evolutionary biology, the cognitive aspect of the cognitive niche refers to the effects of the developmental niche on variations that relate to cognitive functions (Stotz, 2010).

The concept of the cognitive niche we refer to here is a sort of hybrid between the concepts of the selective, developmental, and cognitive niches. However, even though we rely on these parallels to make our argument, a detailed analysis of these is beyond the scope of this paper. The set of questions that fall within our scope relates to the computational function of cognitive extensions and the (developmental and intergenerational) process whereby this computational function emerges. For instance, from an evolutionary point of view, the concept of the cognitive niche that interests us will focus on the evolution of cognitive extensions per se (in a manner akin to cumulative cultural evolution [Mesoudi & Thornton, 2018]).

The niche we consider here is made of niche construction outcomes directly relevant to an organism's activity—for example, extended phenotypes having fitness enhancing impacts (Dawkins, 1982) and “external niche inheritance” such as energetic and informational resources (Odling‐Smee, 2007). External inheritance can secure the reproduction of organisms' life cycle over developmental time—for example, for beaver kits—while causing ecological cascades for other species receiving that inheritance (e.g., through modified communities). We do not include in the cognitive niche outcomes and ecoevolutionary feedbacks that drive evolution by either negatively impacting development (e.g., “negative” niche construction outcomes like feces) or by being “ecological cascades” that can force the exploration of the adaptive landscape (Odling‐Smee, Laland & Feldman, 2003).

The cognitive niche is sometimes studied as a psychological habitat and sometimes as a functional habitat (cf., Bertolotti & Magnani, 2017). The psychological habitat refers to the set of organisms–niche relations that offer organisms relevant action (and perception) possibilities, also known as affordances (Gibson, 1979). The functional habitat is the set of resources that support species specific tasks (e.g., foraging or language and communication in humans [Clark, 2006; Whiten & Erdal, 2012]). This means that one must define the functional habitat on the background of the organism's phenotypic dispositions; for example, books are part of the functional habitats of humans because of humans' ability to read, but they are not part of the beavers' functional habitat. The psychological and functional habitats can be part of the same overall physical habitat. They simply differ in terms of their explanatory scope. The former explains psychological aspects of the organism's experience, such as perception, whereas the latter explains how the organism will rely on the niche to perform some task (e.g., foraging).

#### 1.1.2 Active inference

Contemporary “predictive” theories of cognition include well‐known theories such as predictive coding (Rao & Ballard, 1999), the Bayesian brain (Knill & Pouget, 2004), predictive processing (Clark, 2013) and the predictive mind (Hohwy, 2013), ecological enactivism (Bruineberg, Kiverstein & Rietveld, 2016), and active inference. Active inference, in particular, is commonly used to account for cognitive phenomena such as action, decision‐making, and environmental navigation (Kaplan & Friston, 2018).

Active inference assumes that an organism must entertain minimally uncertain “causal” models—that can generate effects from their causes—of the probabilistic relation between relevant types of events. Uncertainty is an information theoretic notion that relates to Shannon information. Shannon or self‐information can be quantified by measures such as surprisal and entropy. Surprisal *ℑ*(*x*) is a measure of unlikeliness that a random variable *X* takes a value *x*, given a model *m* of how *X* was generated, that is, *ℑ* =  −  ln*P*(*x*|*m*). In turn, entropy *S* = *E*[*ℑ*(*x*)] is the expected or weighted average of surprisal over time. Crucially, the negative of surprisal is also known as log model evidence or marginal likelihood ln*P*(*x*|*m*). This means that minimizing surprisal (i.e., self‐information) corresponds to maximizing model evidence, which has been referred to as self‐evidencing (Hohwy, 2016). Self‐evidencing over time also means minimizing uncertainty or entropy. For instance, an equal probability such as .5 and .5 of observing an outcome (e.g., *X* = {*head*; *tail*}) before any observation (e.g., before flipping a coin) entails a state of full uncertainty (or maximum entropy). The observation of an occurrence (e.g., after having flipped the coin) entails a full disambiguation or maximum information gain. Put another way, one defines the information gained after observing an outcome in terms of the amount of uncertainty that is resolved. Hence, a shorthand for the notion of self‐evidencing is uncertainty reduction. From the standpoint of a physicist, the resolution of uncertainty corresponds to the tendency of lifelike systems to resist the second law of thermodynamics—or strictly speaking, the fluctuation theorems that apply to open systems—by placing an upper bound on their entropy or disorder.

According to active inference, to survive and reproduce when facing environmental stressors, organisms must entertain minimally uncertain models of the relation between sensory inputs they receive (e.g., “scent”) and the possible environmental causes having generated these inputs (e.g., “predator” or “mating partner”). Organisms must also model the probability of transitions among causes in the world (e.g., “predator approaching”) relative to possible actions their physiology permits (e.g., “I can fly” and “I can't swim”). In line with models of Bayes optimal foraging (Okasha, 2013), minimizing uncertainty in such causal, predictive, or generative models involves updating probabilistic mappings or Bayesian beliefs (a.k.a., learning and perceptual inference) and selectively sampling sensory inputs expected under these beliefs (a.k.a., action).

#### 1.1.3 The extended mind

The extended mind approach to cognition (Clark & Chalmers, 1998) claims that cognitive processes can be offloaded to (i.e., reallocated to), or extended through (i.e., transformed into), components that reach beyond the system's internal states (e.g., brain states). The notion of offloading refers to the use of physical action and artefacts to manage the cognitive demand of information processing (for a review, see Risko & Gilbert, 2016). Extended mind theorists suggest that the realization base of some cognitive processes (i.e., states that realize a given cognitive process) come to include reliable, accessible external states of the niche (e.g., the cellphone that functions as extended memory for recalling phone numbers (for a review, see Kirchhoff & Kiverstein, 2019).

### 1.2 Outline

#### 1.2.1 Current limitations

Some have drawn links between the cognitive niche construction perspective and the notion of uncertainty minimization in active inference and implicit self‐evidencing. For instance, simulation studies have shown that by changing the material layout of the niche in a way that mirrors the causal models of the organism, organisms shape their sensory array in a way that is congruent with learned generative models, which entail more efficient reduction of uncertainty over development (Bruineberg, Rietveld, Parr, van Maanen & Friston, 2018).

The mirroring or synchronization that obtains between organisms and their niche has various feedback consequences over evolutionary time. For instance, some proposed that organisms can install in the niche cues that invite action with high epistemic value. Epistemic value relates to the ability of an action to resolve uncertainty—through the selection of actions that solicit the right sort of sensations for resolving ambiguity (e.g., looking under the streetlight or reading an instruction manual, Friston et al., 2015). Through external niche inheritance, salient cues with high epistemic value can be passed on as ecological legacies to guide the epistemic foraging of future generations (Constant, Bervoets, Hens & Van de Cruys, 2020).

The process whereby organisms install epistemic cues in their environment provides a suitable mechanistic account of the notion of instrumental intelligence in cognitive niche theory. However, the mechanics of the functional and psychological dimensions of the cognitive niche remain unexplored in the literature on predictive processing approaches to cognitive niche construction (for interesting discussions of related functions see Bruineberg & Rietveld, 2014; Clark, 2013; Fabry, 2017; Ramstead, Veissière & Kirmayer, 2016).

#### 1.2.2 The argument

In Section 2, we unpack the functional and psychological dimensions of the cognitive niche under active inference. We argue that the cognitive niche—understood as an externally realized cause–effects model—can be modeled as a form of externally realized “shared” generative model that is leveraged and optimized by organisms to perform action‐related adaptive cognitive functions (e.g., decision‐making, navigation, foraging). The optimization and leveraging of this shared generative model, through action and perception, are what we call extended active inference (henceforth, EAI).

We argue that one can study cognitive niche construction under EAI as a bona fide cognitive function in the game of uncertainty minimization, alongside standard functions studied by active inference, such as active sensing and learning. Formally, cognitive niche construction thus construed is geared toward uncertainty minimization, thereby qualifying as a cognitive function under active inference. The functional and psychological aspects of the cognitive niche directly follow from our formalization of EAI (see Figure 2). We conclude Section 2 by presenting two case studies that illustrate the view of cognitive niche construction as a cognitive function.

In Section 3, we explain the relation between EAI, the original approach to the extended mind (Clark & Chalmers, 1998) and the diachronic approach (Kirchhoff, 2012, 2015). When viewed as a cognitive function, cognitive niche construction under active inference allows an epistemological extension of the boundaries of cognition (cf., Kirchhoff & Kiverstein, 2019). Building on Section 2, we argue that the coalition between brain(s) and world that obtains through cognitive niche construction operates through a process of cognitive uploading (Constant, Ramstead, Veissière, Campbell & Friston, 2018).

Cognitive uploading is akin to cognitive offloading in the original theory of the extended mind (Clark & Chalmers, 1998).

However, in contrast to the traditional notion of offloading, the notion of uploading refers to the creation of novel cognitive functions that are taken on board by the cognitive niche per se, instead of being merely managed by the cognitive niche. A function is “offloaded” when individual agents restructure their worlds so as to minimize internal processing costs and/or increase reliability. For example, by posting a yellow stick note on the front door to remind them to pick up milk next time they are out. A function is uploaded when social and technological change means it is now taken care of by the niche rather than the individual. For example, most agents now store their phone numbers using smartphones rather than biomemory. So, the whole “number storage” function (unlike the whole “remember X" function) has been assimilated into the niche. The niche into which the function has been uploaded can then be passed on to future generations for them to leverage, share, and finesse that function.

The original notion of the extended mind applied, in principle, to both these kinds of cases. But the distinction is formally helpful and speaks to different webs of agent‐world dynamics that evolve and alter on different spatiotemporal scales; the notion of offloading speaking to time scales spanning individual‐level dynamics unfolding over real‐time and (neuro)developmental time scales and the notion of uploading speaking to individual‐ and group‐level dynamics unfolding over developmental and intergenerational time scales. Uploading is a stronger species of offloading. EAI formalizes these dynamics as emergent properties of cognitive niche construction. Novel cognitive functions produced through cognitive uploading can result from gene‐culture coevolutionary dynamics that “glue” organisms to those functions performed by the “trusted” niche. Uploading under EAI emphasizes the trade‐off, overevolutionary, and developmental time, of the deployment of on‐board (neuro)biological functions for on‐board (socio)environmental ones, thereby allowing metabolically efficient, though niche bound adaptive behavior that may be favored by selection.

Crucially, cognitive uploading endows external states of the cognitive niche with the ability to track regularities otherwise impossible to track because they are often too complex to be learned by individual organisms. We frame affordances as uploaded proxies that track those complex causal regularities.^1^ Thus, consistent with the theory of diachronic cognition (Kirchhoff, 2015), the notion of uploading can further be viewed as the process whereby agents produce cognitive extensions that gain independence from the specific individuals having produced them. Uploading differs from offloading in that the uploaded cognitive task comes to be shared by other agents. This allows the production of nonindividual specific cognitive extension affording action tracking more complex regularities.

## 2 THE FUNCTIONAL AND PSYCHOLOGICAL NICHES UNDER ACTIVE INFERENCE

Active inference explains perception and learning as processes that conform to an optimization process known as variational inference (Beal, 2003) The motivation for modeling uncertainty minimization in terms of variational inference relates to the sort of perceptual, or rather, inferential challenges faced by living systems such as humans. We have no direct access to the causes of our sensations nor is there a one‐to‐one mapping between causes and sensations (Clark, 2013; Hohwy, 2016; Wiese & Metzinger, 2017), for example, a red sensation might be generated by a red traffic light, a red car, or a red jacket. These kinds of ill‐posed inference problems can only be solved by appealing to prior beliefs or experience to resolve ambiguity or uncertainty; hence, the appeal to schemes such as approximate Bayesian or variational inference.

Variational inference is a ubiquitous mathematical description of (Bayesian) belief updating that describes the formation of perceptual hypotheses that explain our sensations. Variational inference rests on a probabilistic generative model. A generative model is a probabilistic statement about a set of unobserved (hidden) variables (i.e., causes) and observed sensations (i.e., consequences), which represent an organism's predictive or causal model of the world. A generative model is usually expressed in terms of a likelihood and a prior term:

(1) $$\underset{\text{generative model}}{\underset{︸}{p\left(s,\eta \right)}}=\underset{\text{likelihood}}{\underset{︸}{p\left(s|\eta \right)}}\underset{\text{prior}}{\underset{︸}{p\left(\eta \right)}}$$

The likelihood corresponds to the probability of sensations *s* (e.g., “dry” or “wet”) given priors about the state of the world *η* (e.g., “inside a burrow” or “outside a burrow”). The prior corresponds to the probability of conditions, or causes, generating the sensation (e.g., “being in or out of a burrow”), before making a sensory observation. Using variational inference, one can invert the likelihood in Equation (1) to approximate the posterior probability of causes *p*(*η*| *s*) once a sensation has been sampled. This involves the minimization of a bound on the unexpectedness of sensations (a.k.a., surprisal)—called free energy—with respect to the approximate posterior, known as variational density. This density is associated with (i.e., assumed to be encoded by) internal (e.g., brain) states *μ* of the organism:

(2) $$\underset{\begin{array}{l} \text{Free}\;\\ \text{energy of} \end{array}}{\underset{︸}{F}}\overset{\begin{array}{l} \text{sensations and}\;\\ \text{internal states} \end{array}}{\overset{︷}{\left(s,\mu \right)}}=\underset{\text{KLDiv}.}{\underset{︸}{D}}\left[\overset{\begin{array}{l} \text{variational}\;\\ \text{density} \end{array}}{\overset{︷}{q_{\mu }\underset{\begin{array}{l} \text{over}\;\mathrm{ext}.\\ \text{states} \end{array}}{\underset{︸}{\left(\eta \right)}}}}\|\underset{\text{true posterior}}{\underset{︸}{p\left(\eta |s\right)}}\right]\overset{\text{surprisal}}{\overset{︷}{-\ln p\left(s\right)}}$$

In Equation (2), the variational density becomes a posterior belief about the causes of sensations (e.g., “was I in a burrow or outside a burrow *η*, given sensations of wetness *s*”). This inverse mapping—from causes to effects—corresponds to inferring the causes of sensations. In variational inference, approximating the true posterior can be described in terms of minimizing the free energy functional *F*(*s*,*μ*):

(3) $$q_{\mu }\left(\eta \right)=\underset{q}{\arg\min}F\left(s,\mu \right)\approx \underset{\text{inverse mapping}}{\underset{︸}{P\left(\eta |s\right)}}$$

**FIGURE 1:**
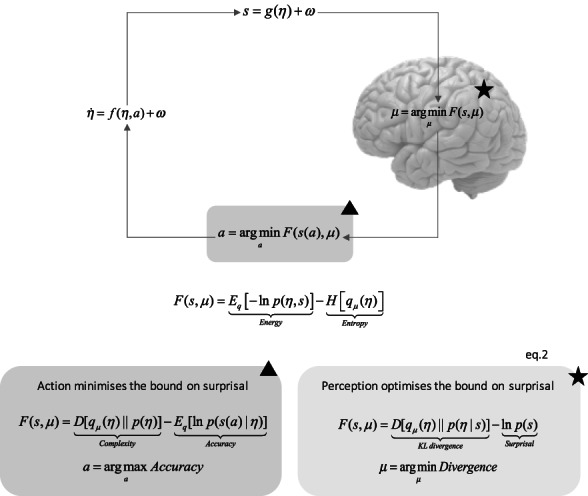
Action, perception, and learning under active inference

In Equation (3), this minimization has two consequences: (i) The functional becomes a tight upper bound on the unexpectedness of sensations (a.k.a., surprisal); (ii) the minimization renders the variational posterior a good approximation to the true posterior. This follows because a Kullback–Leibler divergence *D* is always nonnegative. This means, *F*(*s*, *μ*) ≥  − ln*p*(*s*), with equality when the divergence has been eliminated, *F* =  − ln*p*(*s*) ⇒ *D* = 0 ⇒ *q*_*μ*_(*η*) = *p*(*η*| *s*). Formally, variational inference converts an inference problem into an optimization problem as articulated by Equation (3) (see Figure 1 for a summary).

Assuming that the organism's brain embodies the variational density, variational updates^2^ ensure brain states encode a posterior belief about the true distribution of sensory causes and contingencies in the world, and—by the same token—the organism learns Bayes optimal priors about cause–sensation relationships. This is usually associated with experience‐dependent plasticity (Friston, 2010). Hence, taken together, the dynamics described in Equation (2) explain perception and learning as an optimization process, in which expectations about hidden states of the world and their relationships to each other (and sensations) are minimized with respect to free energy.

This optimization unfolds over several timescales. Neurophysiological states that underwrite inference changes quickly (on a timescale of milliseconds). Neuronal connections that learn contingencies change over minutes to hours, via experience‐dependent plasticity. Finally, the functional architectures that entail the generative model change over a neurodevelopmental timescale of months to years, as the phenotype becomes a sufficiently good model of its (encultured) cognitive niche (compare with the good regulator theorem [Conant & Ashby, 1970]).

Finally, in active inference, organisms are viewed as possessing priors about expected or preferred outcomes of action. This simply means that actions are selected if they bring about expected outcomes, while being geared toward minimizing expected surprise (i.e., uncertainty) about the future (Friston et al., 2014). Hence, in active inference, motor (and autonomic) functions work hand‐in‐hand with a perceptual inference to resolve uncertainty through the active sampling of salient, uncertainty reducing sensations, while allowing for preferred, unsurprising outcomes (green box, Figure 1).

The basic formalism corresponds to optimizing a free energy functional of sensations and expectations encoding beliefs about hidden states of the world *F*(*s*, *μ*). This functional can be expressed as energy minus entropy—by analogy to free energy in statistical physics. Various rearrangements of the free energy functional can be used to formalize various cognitive phenomena, namely, action in the green box (triangle indicator) and perception in the purple box (star indicator). Upper panel: Sensations *s* and action *a* are the quantities that couple internal states' *μ* to external, hidden states in the environment *η*. The argmin operator refers to variational updates—for an introduction to variational inference in relation to other inference schemes (e.g., expectation maximization) algorithms (Beal, 2003). External states are described in terms of equation of motion that includes random fluctuations *ω*. Purple box: Perception optimizes internal states. The mathematical formulation of free energy corresponds to Equation (3) in the text. Green box: Action minimizes the free energy bound by increasing the accuracy of sensations, for example, by selectively sampling expected sensations. Note that action does not consider posterior beliefs in the Kullback–Leibler divergence. This reflects the fact that action can only change free energy by changing sensory inputs. When choosing among different actions, the free energy is minimized with respect to “counterfactual” outcomes by taking the expectation of free energy, under future outcomes, given the action being evaluated. In this instance, maximizing expected accuracy is equivalent to minimizing ambiguity. Similarly, minimizing expected complexity minimizes risk, defined as the divergence between predicted and preferred outcomes.

### 2.1 The cognitive niche

Changes in brain states and functional architectures optimize organisms generative (i.e., causal) model of the causal structure of their cognitive niche. Interestingly, one can use the variational formalism to model and study changes in an environment, or external states, in the same way one does for experience‐dependent learning in the brain (Bruineberg et al., 2018; Constant et al., 2018). We now show how this formal symmetry yields a view of cognitive niche construction as a form of environmental “learning” about the organisms hosted by the environment. On this view, organisms effectively “teach” the environment what actions they should expect (i.e., construct externally realized causal models of the effects of action—where action, from the point of view of the environment now becomes a sensory datum).

The environment is the generative process that is modeled by the generative model entailed by the phenotype. However, in virtue of the mathematical symmetry imposed by a Markov blanket (that separates internal and external states) (see Clark, 2017; Friston, 2013; Kirchhoff, Parr, Palacios, Friston & Kiverstein, 2018; Ramstead, Badcock & Friston, 2018), the environment can also be construed as a generative model of its denizens, who now becomes the processes generating outcomes for the environment. In other words, the external or environmental states play the dual role of generating outcomes for organisms, while also encoding probabilistic “beliefs” about organismal processes. We will see that one can treat the environment as inferring the cause of the “sensations” it receives from being acted upon by its denizens.

**FIGURE 2:**
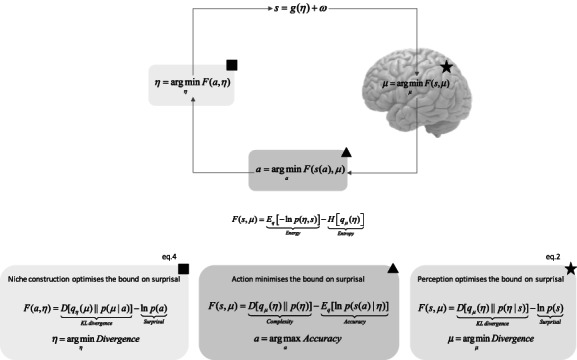
Cognitive niche construction and extended active inference

We do not claim that the formal symmetry between brain and niche dynamics entails a symmetry in construal. Rather, we employ the notion of symmetry epistemically, as a modeling “analogue” (cf., Figdor, 2018), to make sense of niche dynamics as learning dynamics under active inference. The notion of symmetry is merely an assumption that allows us to write the formal model (Figure 2) presented in this section. The added value of our model, as it pertains to this paper, is to provide a mechanistic basis for the psychological and functional aspects of the cognitive niche. The model on offer is readily implementable in silico simulations of active inference, thereby yielding potential novel avenues for empirical research on cognitive niche construction and extended cognitive science.

Formally, what counts as a sensation in the environment are the physical actions of organisms. Then, causes of sensations can be modeled as the priors of the organism having given rise to action (i.e., niche sensations) (Ramstead, Constant, Badcock & Friston, 2019). Just as for the photon that hits the retina—thereby generating a sensory input leading to Hebbian learning in the brain—one can model the action of the organism encoding traces of behavioral regularities in the environment. What counts as Bayesian priors in the environment are the probability mappings between action and the organism's prior about action (Figure 2). Effectively, this closes a circle of causality, in which the niche and phenotypes are trying to learn about each other to minimize their joint free energy or surprise. An inevitable consequence of this is that the niche and its incumbents become mutually predictable—in both directions of fit—so that the joint niche‐phenotype system can be regarded as jointly self‐evidencing.

Take for instance the phenomenon of desire paths. Pedestrians often leave traces in parks as they cut through the grass on their commute. Over time, these traces might become deeper, thereby telling newcomers this trail is likely to lead to outcomes preferred by the people having carved the paths; namely, people like me, who prefer or predict the same sorts of things. In so doing, desire paths encode mappings between possible actions and outcomes (e.g., “if I follow this path, I will find the café”). These mappings can have different degrees of reliability. At first, they may be ambiguous, as multiple shallow traces may encode different alternative action‐outcome mappings of equal prior probability *p(μ|a)* (e.g., “this path may take me to the café”). As a path becomes more salient, it will further attract pedestrians who desire to cut through the park to reach the café, which will further consolidate the trail. Over time, assuming that people indeed find the café, the path will encode traces reducing uncertainty about the way to the café.

By analogy to perception and learning in Equation (2), one can formalize cognitive niche construction as a minimization of free energy from the point of view of the niche (see also Figure 2):

(4) F︸Freeenergy ofaη︷organisms'actions&states of the niche=D︸KLDiv.qημ︸beliefs aboutorganisms'internal states︷variational density‖pμa︸true posterior oforganisms'internal statesgiven action−lnpa︷affordance

Equation (4) has the same form as Equation (2) but with internal (sensory) and external (active) states switched around. This means that the variational density *q*_*η*_(*μ*) is taken under the external states *η*, not internal states of the organism *μ*, and surprisal is relative to organisms' actions. Equation (4) shows that casting changes in environmental states as self‐evidencing makes the variational density—encoded by the states of the niche—a good approximation to the posterior probability over the internal states of its organisms, having observed their actions. Put another way, under this extended form of self‐evidencing, the material layout of the niche will look as if it “learns” about organismal “beliefs” causing preferred action, in the same way as organisms' learn about environmental causes generating sensations.

Clearly, we are not limiting this interpretation to desire paths; in principle, any aspect of the niche can be subject to this interpretation—including cognitive, cultural, and any other deontic states of the world, that is, states that tell an agent what action to select (Constant, Ramstead, Veissière & Friston, 2019). Language itself may be considered as a kind of meta‐level niche‐construction—a tool that allows the rapid emergence and adaptation of locally relevant niches (Lupyan & Clark, 2015)—as when someone says “the café” is under the awning across the street.

As in Figure 1, internal states and action change to minimize free energy based on sensations and internal states. Coincidentally, antisymmetric processes unfold in the niche. The key point in the figure is that all the quantities in the purple box that describe internal dynamics are inverted in the beige box—describing niche (i.e., external) dynamics. From the point of view of the niche, the action of the organism *a* is a “sensation,” sensations of the organism *s* are “actions,” and internal states of the organism *μ* are “external states.” Beige box (square indicator): Cognitive niche construction as environmental “teaching” makes the environment's free energy a bound on environmental surprisal. Environmental surprisal here is the unexpectedness of an organism's action—or the negative log probability of encountering a particular action. This can be read as a mathematical description of affordance. In bounding surprisal, the variational density of the environment ends up reflecting the most probable states of the organism, given that organism's behavior. The expression in the beige box is reproduced in Equation (4).

### 2.2 The psychological niche

As mentioned in the introduction, proponents of the psychological niche view the niche as a set of affordances (Rietveld & Kiverstein, 2014). In our model, the niche's free energy bounds the surprisal of an organisms' action and therefore can be viewed as an evidence bound on the probability of an observed action, averaging over an organisms' priors and preferences.^3^ As expressed in Equation (4), changes in the physical states of the niche (e.g., the production of niche construction outcomes) will optimize a bound on the surprisal of organisms' action, which corresponds to the (negative) affordance of an action on the environment. By analogy with the creature‐centric formulation of free energy, affordance just is the (log) evidence provided by an action for the niche's generative model of the active creatures it is trying to learn about.

Modeling niche's dynamics with the formalism in Equation (4) allows us to derive a formal notion of affordances that are built into the variational formalism. Our formal interpretation supports the view according to which affordances are organism‐specific action probabilities (Bruineberg & Rietveld, 2014; Tschacher & Haken, 2007) whose gradients drive niche construction, via a joint (i.e., extended) minimization of variational free energies. Importantly, our model clarifies the manner in which the concept of affordance may be implemented in in silico simulation studies and empirical research under active inference, as it makes this notion readily implementable with the freely available simulation routines employed in active inference research (see the various DEMOs of the statistical parametric mapping 12, MATLAB toolbox at, fil.ion.ucl.ac.uk/spm/software/spm12/). Artificial data acquired from in silico simulations of affordance production and leveraging could then be compared with empirical data (cf., Cullen, Davey, Friston & Moran, 2018; Mirza, Adams, Friston & Parr, 2019) to test hypotheses about EAI as an emergent property of cognitive niche construction under active inference (e.g., in a foraging or navigation task).

The notion of extended active inference or self‐evidencing reflects the extensive aspect of free energy; namely, the free energy of two systems (i.e., organism and niche) is just the sum of their respective free energies, conditioned upon the (i.e., sensory and active) states they share (Bruineberg et al., 2018). The psychological niche can thus be viewed as a state space of invitations to act, with peaks and valleys that correspond to the most and least probable (and thereby adaptive) actions, given the priors and phenotypic preferences of organisms “like me” having constructed the niche in first place.

### 2.3 The functional niche

Active inference assumes that cognitive functions are in the game of optimizing an organism's generative model about the cause of its sensations. This amounts to minimizing free energy or maximizing model evidence through variational updates (i.e., perception—purple box Figure 1), and to the selective sampling of expected sensory information (i.e., action—green box Figure 1). We now argue that cognitive niche construction (beige—box Figure 2) can be framed as a cognitive process, as construed by active inference, that optimizes an organism's generative model vicariously as part of an extended process of self‐organization or self‐evidencing. Niche and organisms can be meaningfully studied as trying to optimize their respective models of each other.^4^

The take home message of Section 2 is illustrated in Figure 2; namely, one can study the niche as the organism's generative process or a generative model of the organism—in the sense of Tooby and DeVore (1987, p. 2010)—that implicitly learns about organismal priors and preferred behavior. This explains why resources encoded by acting on the functional niche come to cue or afford adaptive action. As argued above, resources in the cognitive niche cue actions that were selected by conspecifics in the past. Once learned, cues—conveyed as affordances—gear the organism toward selecting actions that will tend to be adaptive (more often than not), relative to the task that entailed the carving of the niche in first place. Task specific, adaptive actions thus just are actions that bring about sensory information that are expected under the sort of priors and preferences that constitute the phenotype of organisms “like me” (Constant et al., 2018; Friston, 2010).

### 2.4 Case study

In this subsection, we unpack the view of cognitive niche construction as a cognitive function through a well‐known case study in niche construction theory: The phylogeny of freshwater kidney in common earthworms (*Lumbricus terrestris*). We take this case study as an illustration of the way earthworms optimize their generative model by encoding reliable cause–effect relationships in their environment. We then provide some examples of the effect of cognitive niche construction as a cognitive function in humans by focusing on a discussion of spicing in food preparation.

Common earthworms are phylogenetically related to aquatic freshwater worms. Freshwater worms have kidneys that remove excess water from their body. This trait is consistent with aquatic environmental conditions but far from being adaptive for terrestrial life conditions, as water is limited, and water conservation should be the norm. Thus, all things being equal—in the world of natural selection—common earthworms should have evolved water‐balance organs that favor water conservation. However, common earthworms still have roughly the same freshwater kidneys as their ancestors. A plausible explanation for this is that the niche construction undertaken by earthworms might have tipped the balance in evolution. By constructing—and inheriting—semiaquatic environments like moist soils, common earthworms might have softened selection pressures on water‐balance organs (Satchell, 1983; Scott Turner, 2009). Put another way, the niche became part of common earthworms' solution space to the challenge of having water removing organs in dry environments. The niche then allowed economies of “evolutionary money” to be spent on biological adaptations (e.g., selecting for water conserving organs), thereby explaining, in part, the evolutionary trajectory having led to the current phenotype.

In the parlance of active inference, the niche of common earthworms functions to inform a predictive (or generative) model of the relation between states of the world (e.g., “in a burrow” or “outside a burrow”) and sensory outcomes (e.g., “wetness” or “dryness”), cueing earthworms about relevant cause–effect relationships (Christopoulos & Tobler, 2016). The networks of burrows that generations of earthworms constructed (and inherited) came to afford adaptive action in the sense that engaging them most likely led to locations affording a priori preferred level of wetness. In other words, cognitive niche construction outsourced the computation of adaptive action to the environment per se. Calling on recent numerical analyses and theoretical treatments of active inference in decision‐making, we speculate that a consequence of this is that earthworms could simply rely on the action afforded by the niche to avoid computing action that would fulfil their evolutionary (prior) preferences for wet soil, which would soften selection on water balance organs.

Cognitive niche construction here operates through (i) the increase in performance enabled by the outsourcing of the computation to the niche and (ii) the absence of an adaptation due to niche construction. First, constructing cognitive niches so as to make them more predictable (i.e., navigable) enable the organism to reduce model complexity^5^ by constraining the variety of sensory causes that the organism has to entertain (Sengupta, Stemmler & Friston, 2013). This allows the enhancement of performance for exploitative, fitness‐related behavior (Friston et al., 2016). Indeed, tracking the potential causes of sensations in a constantly fluctuating world is costly as it requires to entertain multiple counterfactual priors (e.g., “will I end up in a wet environment if I move left, right, up and, down, etc.?”). Outsourcing the computation of these counterfactuals to the niche can be expected to increase performance in terms of both thermodynamic and inferential efficiency. Second, the enhancement of performance may be reflected in more efficient reaction times during exploitative behavior, which would favor the reproduction of a phenotypes that call on the predictability afforded by the niche.^6^

In earthworms, the circular causality over developmental and evolutionary time scales between the optimization of generative models through environmental modifications and the coupling to those environmental modifications over evolutionary time may explain the softening of selection on things like water‐absorbing organs. This may be viewed as a form of developmental constraint on selection, that is, the strategy of outsourcing the computation became locked‐in, because of the advantaged it provided, yet, to the cost of a phenotype that would heavily rely on this strategy (e.g., a phenotype that would not possess the right kidney). The phylogenetic trajectory of earthworms exemplifies the phenomenon of cognitive uploading discussed in the introduction of this paper. Uploading saves metabolic resources through the reliance on epistemic cognitive extensions that are typically internally realized. Over multiple generations, this comes at the cost of becoming “evolutionarily glued” to those cognitive extension. Put bluntly, cognitive niche construction smartens the world of the earthworm, so that its physiology can remain dumb yet optimal in peace (Clark, 1998).

The example of the earthworms speaks to the fact that characteristic behavioral patterns or components of phenotypes (extended or else) will emerge from the construction of the cognitive niche and its impacts on evolution and development. Cognitive uploading could also allow one to formalize the computational architecture of the human phenotype. For instance, the inheritance of epistemic resources over evolutionary time and the re‐enactment of the practices invited by these resources over development underwrites the phenomenon of tradition, understood as learned a new behaviors supported by sociocultural practices (Fragaszy & Perry, 2003). In humans, traditions and associated artefacts undergo processes of cultural evolution (Boyd & Richerson, 1988), which enables intergenerational groups to converge on adaptive repertoires of tools, technologies, rituals, and so forth, that have been filtered by generations of conspecifics (for a review, see Laland, 2018).

Evolved traditions enable the success of complex cognitive tasks, while leaving the structure of the causal models to which the success of these tasks relate unbeknownst to the agent (Fragaszy, 2011). In his book *The secret of our success* (Henrich, 2015), Harvard anthropologist Joseph Henrich provides a series of such simple examples in which traditions track cause–sensation relationships, otherwise impossible to track thereby securing adaptive low‐cost behavior. One such example is the use of spices in food processing. Spices generally have no nutritional value and are often made of aversive active ingredients. Yet, many humans use them abundantly because some of those active agents turn out to kill foodborne pathogens present, for instance, in widely consumed food like meat; something that is generally unknown to people having acquired and reproducing the practice, yet that is highly beneficial to them. Traditions of spicing per se come to model hidden causes whose structure could not be discovered by individuals alone over their lifespan. In the spirit of Henrich's reflection, culture makes us smart.

From the point of view of cognitive niche construction as a cognitive function under active inference, spicing traditions are intergenerational group‐level strategies to track the complex multidimensional causal relationship between spices, active agents, foodborne pathogens, and meat consumption behavior, which supports the reproduction of the behavioral phenotype. Spicing traditions thus can be viewed as encoding a generative process constructed by multiple generations about what compound is deleterious to what pathogen and what pathogen is deleterious to humans and what spices should be consumed. Enculturated agents, then, become coupled to this generative process which secures adaptive food processing.

Crucially, it is the generative process embodied by the tradition per se that tracks this complex causal relationship, not individual agents. In responding to affordances (a.k.a., epistemic cues of least improbable action engaged by conspecifics; cf., Figure 2) such as those offered by artefacts of traditions, organisms like us manage to succeed implicitly in tasks for which causal models are too complex and too costly to be taken on‐board. Tradition endows individuals with the ability to read into deep hidden causal regularities. In a scaffolded fashion (cf., Sterelny, 2010), the structure of extended cognition is explained formally in terms of intergenerational learning dynamics in the generative process produced by generations of niche constructing agents (i.e., people participating and reproducing the tradition) and by the enculturation of individuals' generative models through the learning of the epistemic cues (a.k.a., affordances) in the generative process.

## 3 EXTENDED ACTIVE INFERENCE

Over developmental time, smartening the world through cognitive niche construction operates through processes akin to that of cognitive offloading, such as studied by the extended approach to cognition. From the perspective of active inference, cognitive niche construction brings the notion of offloading a step further. As we have seen with the earthworm and food preparation examples, cognitive uploading through cognitive niche construction entails outsourcing the inference over future outcomes to epistemic cues of the niche (a.k.a., affordances). Thus, through niche construction, organisms manage to upload self‐evidencing processes directly to the structure of the generative process.

Uploading entails more than relying on physical action and artefacts to support, or help carry out, cognitive functions. The evaluation of expected surprise drives action selection. Self‐evidencing refers to the process of minimizing the bound on surprisal (a.k.a., negative log model evidence) through perception (optimizing the bound) and action (minimizing the bound) (cf., Figure 1); hence, cognitive uploading through cognitive niche construction outsources part of the computation of self‐evidencing processes (those relating to action). Put simply, cognitive uploading helps agents to minimize the bound on surprisal.

In the remainder of this paper, we explain the manner in which the above formalism grounds EAI and generalizes two varieties of claims on extended cognition, the original approach to the extended mind (Clark & Chalmers, 1998) and its recent reinterpretation as diachronic cognition (Kirchhoff & Kiverstein, 2019). We show how EAI supports the theory of the extended mind by providing mechanistic explanation of well‐known concepts such as the parity principle, functional isomorphism, epistemic action, and diachronic cognition. We do not engage the many debates surrounding the varieties of extended cognition. This is well beyond the scope of this paper. Rather, the hope is to provide future researchers with a formal apparatus to make progress in these debates by showing how the varieties of claims on extended cognition may be formally expressed in EAI, a lingua franca of sort such as summarized in Figure 2.

### 3.1 The extended mind under EAI

#### 3.1.1 Parity principle under EAI

The original theory of the extended mind decomposes into three features. The first is a parity principle. The role of the parity principle in the theory of the extended mind is to first help us to conceive of the view of the mind as being extended into external vehicles; the parity principle is “a mean of freeing ourselves from mere biochauvinistic prejudices” (Clark, 2005, p. 2). The parity principle states that:

If … a part of the world functions as a process which, were it done in the head, we would have no hesitation in recognizing it as part of the cognitive process, then that part of the world is … part of the cognitive process. (Clark & Chalmers, 1998, p. 8)

If we agree that the function performed by an external state during a cognitive task would qualify as a bona fide cognitive function “were it done in the head,” then that external state in question ought to be considered as potentially an integrative part of the cognitive architecture of the cognitive system. This principle is vindicated by the formalism of EAI presented in this paper; as we have shown, the description of the dynamics underlying learning in the generative process are formally equivalent to the learning in the generative model. Of course, one must consider the part of the generative process that is coupled to the generative model through cognitive niche construction.

#### 3.1.2 Functional isomorphism under EAI

The parity principle entails the second feature of the theory of the extended mind, which is the notion of a potential functional isomorphism between some internal and some external states (Sutton, 2010). Functional isomorphism stresses that internal and external states have to be seen as equivalent with regard to the basic properties of cognition. For instance, under certain conditions, a notebook might very well play the same coarse‐grained functional role or epistemic function than biological memory implemented by patterns of neuronal connections in the brain. When looking for coarse‐grained parity between internal and external cognitive resources, it has been suggested that external resources should meet the requirements of “glue and trust” so that the resource is available when needed (like biomemory) and not subject to constant agentive scrutiny—to ensure it is working as it should (again, like biomemory).

From the point of view of EAI, the trust condition is guaranteed by the uploading process whereby the agent learns to engage epistemic cues of the generative process. This entails trading‐off on‐board neurocognitive functions for on‐board environmental ones. The benefit is the increased performance, though at the cost of increased dependence on the environment. The glue condition is guaranteed by the increased performance that underlies the uploading. For instance, the earthworm is “glued” to its inheritance of burrows and moist soil because of the constraints burrows, and soils have operated on earthworm's phylogeny. We can imagine how an individual would become “glued” to her environment in a similar fashion, though over developmental time scale. For instance, we can imagine an individual that would carve out a path on her commute and, over time, come to heavily rely on that path to arrive to the office on time. The short cut may free up her schedule enough for her to get use to stop at the café to grab a quick espresso during her commute. Then, the individual might stop buying coffee for her kitchen; this would surely simplify the planning of her weekly stop at the grocery store anyway. This, however, would come at the cost of sticking to her path and the espresso it affords. In this hypothetical scenario, the trade‐off that glues the individual to her environment is instantiated by the acquisition of a habit whose robustness relies on (un)learned states of the generative model and learned states of the generative process.

#### 3.1.3 Epistemic action under EAI

The original theory of the extended mind argues that the environment on which cognitive agents rely enables them to perform epistemic actions (Clark & Chalmers, 1998). Epistemic actions are defined as actions that ease or optimize cognitive tasks by reducing the memory load required to perform a task (space complexity); by simplifying the computational processing procedure (time complexity); and by minimizing the probability of error outcomes (success probability) (Kirsh & Maglio, 1994). A notebook, for instance, can be viewed as supporting, and easing the task of, say, making it to your multiple appointments throughout the week, as it will encode relevant information like addresses (i.e., save on space complexity), provide a structure like a schedule for knowing when your appointments are, and how best to coordinate them (minimizes time complexity), and will probably increase your chances of making it on time (increase success probability). These intuitions are formalized by the process of uploading from the point of view of EAI but, in addition, by accounting for the relation between all these advantages. Space complexity corresponds to reduced numbers of counterfactual scenarios that one has to model, which naturally entails minimizing the probability of errors (i.e., the more complex the generative model is, the more likely it is to overfit), and by the same token increases performance in terms of time complexity of computation.

Another (complementary) way to view the picture of extended minds under EAI is to note that neutrally supported estimations of salience (a.k.a., expected surprise) help select actions that can purposefully roll in cognitive operations flowing through bioexternal resources. That rolling in can be internally instigated (e.g., as when I retrieve my smartphone to ensure I do not miss my flight). My purposeful rolling in can also be cued by the external resources themselves (e.g., if I set an alarm for 2 hr before the flight). In that case, the drive or readiness to act to minimize my uncertainty (or to increase the precision of my beliefs about the time) will reduce, as my expectation about future surprise or salience will decrease (e.g., “I will not feel the urge to keep verifying the time at short intervals because I will know when to access my phone”). Here, salience is managed by the cell phone, as trustworthy information is made reliably available. Crucially, the internal flux of precision (i.e., uncertainty in my beliefs) is resolved by the externally structured flow of epistemic (i.e., salience minimizing) action that serves to improve the long‐term fit between my actions and my goals, as well as the cost of computing these long‐term goals. Temporary coalitions of internal and external resources are thus recruited in the same way as are temporary purely inner coalitions, which likewise emerge as varying patterns of effective inner connectivity controlled by fluctuating precision and salience estimations (see Clark, 2015, Chapters 8 and 9).

As we will see below, both the long‐term built environment and the cultural milieu further scaffold this process, nesting our individually extended minds inside larger co‐constructed niches that likewise extract, flag, and cue optimal (i.e., expected free energy minimal) action.

#### 3.1.4 Diachronic cognition under EAI

The diachronic perspective casts cognitive systems as extended, not only in terms of their spatial realization, beyond the spatial scales at which the agent exists, but also in terms of its temporal realization, to (legacy) scales that cognition occupies historically, and in the context of cultural practices in the here‐and‐now. Cognitive assemblies are formed and maintained diachronically, beyond the local organism‐centered boundaries of individuals (Kirchhoff, 2012, 2015, 2018; Malafouris, 2015; Stotz, 2010). Cognitive assemblies are decentralized systems, or networks of human‐and‐nonhuman agencies (Latour, 1993), whose causal constitutive relationship depends upon self‐organized processes distributed across the network they constitute (cf., Figure 2 for a simple environment–organism system).

The standard example used to explain diachronic cognition is that of the Elizabethan theatre companies (Tribble, 2005). Tribble explains how players of the Elizabethan theatre companies during the 16th century would manage to perform multiple different plays per week without being able to rehearse due to time limitations. The ability of the actors to memorize how to perform plays depended on patterned sociocultural practices mediated by material artefacts populating the stage (e.g., stage doors, playing platform, plots, and scripts) and a cross‐generational apprenticeship system (Sutton, 2010) allowing the (re)acquisition of the skills necessary to leverage the informational structure afforded by the augmented stage.

Under EAI, this allows the environment to learn shared preferences and narratives under the form of epistemic cues but only to the extent they are preserved by organisms acting on that environment. Each member of the theatre company engages the diachronic assembly as a generative process from the stance of their generative model. For each individual, other people and artefacts come to encode affordances that indicate what action will be successful because of the ongoing uploading of epistemic cues to the generative process through the apprenticeship practice. As in the earthworm case study discussed above, learning how to leverage these cues allows each individual to limit the complexity of their generative model, thereby enhancing performance (e.g., memory recall, reaction times, etc.) and allowing patterned, low‐cost action selection.

## 4 CONCLUDING REMARK

The model of cognitive niche construction proposed in this paper offers a formal apparatus for the study of non–brain‐based factors in cognition. This paper argued that cognitive niche construction could be viewed as a bona fide cognitive function. Then, we sketched some examples of how this model could be used to give a formal grip to theories of the extended mind and diachronic cognition.

The point stressed in this paper was that cognitive niche construction can be studied as a shared cognitive function enabling organisms to track—often implicitly and at low cost—cause–effect relationships otherwise difficult, if not impossible to track; notably, relationships wherein the hidden causal structure is highly volatile or wherein the hidden causal structure is too complex to be learned solely based on sensations available to the biological sensory apparatus of a single phenotype. From the point of view of extended active inference, all cognitive functions are in the game of tracking causal regularities, and there is no principled reason to restrict this process to the boundaries of skin, skull, or even individual agents.

## Footnotes

^1^
Note that here we are concerned with a Gibsonian notion of affordances understood as action possibility directly perceivable in the environment. For a discussion of niche construction and pragmatic and epistemic affordances relative to mental representation of action—for example, Cisek (2007) and Friston et al. (2012)—see Linson, Clark, Ramamoorthy and Friston (2018).

^2^
Variational updates are a ubiquitous form of Bayesian belief updating. In this paper, “beliefs” are used in the sense of belief updating and belief propagation; namely, non‐propositional probability densities.

^3^
Mathematically, model evidence is also known as a marginal likelihood. This is because the evidence involves an averaging or marginalisation over the causes of some data; here, the datum is the action of an organism that is sensed by the niche.

^4^
It might be argued that as this process unfolds, brains really do (due to their telos) alter so as to fit the world but that it merely appears as if the world alters so as to fit the brain. If I press my punch into the wax, it may seem odd to depict the wax as actively modelling my punch. However, if I consider the wax in relation to my hand, my hand in relation to the letter, the letter in relation to the mailman, the mailman in relation to the postal service, and the postal service in relation to my friend to whom my sealed letter is destined—all of which, just like the wax, are external states to my brain, attributing to that entire ecology the ability to engage in something like active modelling—as well as a deep hierarchical structure—starts to become more tenable (Ramstead, Kirchhoff, Constant & Friston, 2019). The point here is that neither of the internal or external (sub)components of the brain‐wax system exist in isolation. “Oddness” arises when considering the wax as isolated from its embedding, just like oddness would arise from considering the motility of a single dendrite in isolation from the rest of its neural ecology. We do not have more space to elaborate on this argument. All that matters for our current purposes is the availability of an essentially symmetric formalism within which to model processes of mutual modelling between agents and their niche, which reflects genuine, relevant, and perhaps more easily conceivable forms of mutual adaptation.

^5^
Complexity here, is used in the technical sense of statistical complexity or complexity cost. Model evidence (i.e., negative free energy), is expressed as accuracy minus complexity. This means that self‐evidencing is necessarily optimized when accurate model predictions are maintained with minimum complexity (see Equation (1)).

^6^
Technically, this is expressed in terms of a variational principle of least action. In other words, the imperative for self‐evidencing is to minimize the time average of free energy, where this time average is known in physics as an action (not to be confused with the action associated with acting on the niche).
